# A Randomized Clinical Trial of Auricular Point Acupressure for Chronic Low Back Pain: A Feasibility Study

**DOI:** 10.1155/2013/196978

**Published:** 2013-02-28

**Authors:** Chao Hsing Yeh, Lung Chang Chien, Devora Balaban, Rebecca Sponberg, Jaclyn Primavera, Natalia E. Morone, Ronald Glick, Kathryn M. Albers, Susan M. Cohen, Dianxu Ren, Li Chun Huang, Lorna Kwai-Ping Suen

**Affiliations:** ^1^School of Nursing, University of Pittsburgh, 3500 Victoria Street, 440 Victoria Building, Pittsburgh, PA 15261, USA; ^2^Department of Internal Medicine, Washington University in St. Louis, MO, USA; ^3^Department of Medicine, Division of General Internal Medicine, University of Pittsburgh School of Medicine, VA Pittsburgh Healthcare System, Geriatric Research Education and Clinical Center, Pittsburgh, PA, USA; ^4^School of Medicine, Departments of Psychiatry, Physical Medicine, and Rehabilitation, and Family Medicine, University of Pittsburgh Medical Center, Pittsburgh, PA, USA; ^5^Department of Medicine, University of Pittsburgh, Pittsburgh, PA, USA; ^6^World Academy of Auricular Medicine, Hoover, AL, USA; ^7^School of Nursing, The Hong Kong Polytechnic University, Hong Kong

## Abstract

*Objectives*. This prospective, randomized clinical trial (RCT) was designed to investigate the feasibility and effects of a 4-week auricular point acupressure (APA) for chronic low back pain (CLBP). *Methods*. Participants were randomized to either true APA (true acupoints with taped seeds on the designated ear points for CLBP) or sham APA (sham acupoints with taped seeds but on different locations than those designated for CLBP). The duration of treatment was four weeks. Participants were assessed before treatment, weekly during treatment, and 1 month following treatment. *Results*. Participants in the true APA group who completed the 4-week APA treatment had a 70% reduction in worst pain intensity, a 75% reduction in overall pain intensity, and a 42% improvement in disability due to back pain from baseline assessment. The reductions of worst pain and overall pain intensity in the true APA group were statistically greater than participants in the sham group (*P* < 0.01) at the completion of a 4-week APA and 1 month followup. *Discussion*. The preliminary findings of this feasibility study showed a reduction in pain intensity and improvement in physical function suggesting that APA may be a promising treatment for patients with CLBP.

## 1. Introduction

Chronic low back pain (CLBP) is the most prevalent musculoskeletal condition that individuals seek treatment for; thus, it places an enormous burden on societies and healthcare systems around the world in terms of healthcare costs, work and school absenteeism [1–3]. The prevalence in adults over the last 3 months has been reported at 23%, with 11-12% of those experiencing disability due to their back pain [1, 4]. Although hundreds of studies of new interventions have been conducted in the past decade [5], the magnitude of CLBP related problems is expected to continue increasing in the United States and worldwide [5–7]. Different strategies (i.e., education, exercise, simple analgesics, spinal manipulation, mobilization, massage, and acupuncture) have been suggested as reasonable approaches to CLBP [5], but these treatments have limited efficacy [5]. Analgesic use is also limited by a variety of adverse side effects, including drowsiness, constipation, dry mouth [8], and the potential for addiction [9]. The increasing prevalence of pain and ineffective CLBP-management highlight the limitations of current pain management strategies.

Acupuncture has shown promising effects for low back pain treatment [10–13]. However, the widespread application of acupuncture to manage pain is limited by the lack of compelling evidence from high-quality clinical trials [14], the need for patients to travel to the acupuncture site frequently [15], and the cost of the acupuncture treatments not being covered by insurance [16]. Thus, the application of an acupuncture-like stimulation to ear acupoints (auricular point acupressure, APA) is a potential solution for the unmet pain and cost challenges for CLBP.

APA is a form of auricular therapy based on Traditional Chinese Medicine (TCM) that uses acupoints on specific areas of the inner and outer auricle to treat a disease/illness, and has been part of TCM for more than 2000 years [17, 18]. The World Health Organization considers auricular therapy a form of microacupuncture that can affect the whole body [19]. Unlike acupuncture, APA requires small objects (e.g., botanical plant seeds or metal, magnet pellets, approximately 2 mm in size), applied to the patient's ear acupoints with a small piece of waterproof tape (usually 8 × 8 mm) [20]. Once applied by a trained therapist, participants can self-manage at home by applying pressure to the seeds and thus requiring fewer office visits. APA has shown promising analgesic effects in dysmenorrheal [21–23], postoperative pain [24–26], hip fracture [27], low back pain [28, 29], and pain from bone marrow aspiration [30]. Our preliminary open-pilot trial of APA found that 64 participants who received 1 week of APA for CLBP reported a 46% reduction in their “worst pain,” 54% reduction in their “average pain,” and a 56% reduction in their “overall pain severity” after 7 days of APA treatment [20]. Given our promising findings of 1-week APA, we wanted to examine the feasibility of a 4-week APA protocol for CLBP to determine if longer treatment could achieve greater improvement.

## 2. Materials and Methods

This prospective, randomized clinical trial (RCT) feasibility study was designed to investigate the effectiveness of APA in the management of CLBP. Participants were randomized to two groups: true APA (true acupoints with taped seeds on the correctly designated points for CLBP) or sham APA (sham acupoints with taped seeds but on different acupoints than those designated for CLBP). Duration of treatment was four weeks. The data assessment included six in-person time points (baseline, 4 week, and 1-month followup). Participants also filled out a daily diary that included questions on pain intensity, back-specific dysfunction, medication use, and adverse events.

### 2.1. Participants and Setting

Participants were eligible for the study if they (1) were of age 18 years or over; (2) were able to read and write in English; (3) had CLBP defined as low back pain of at least three month duration; (4) were willing to commit to weekly study visits for 4 weeks and two followup visits (at completion of treatment and at one month after treatment); (5) reported an average pain intensity score related only to their CLBP of ≥4 on a 0 to 10 point numerical pain scale in the past week. Participants were excluded if they had (1) malignant, autoimmune disease or recent trauma causing their pain; (2) concurrent use of other adjunctive pain therapies (i.e., physical therapy, chiropractic treatment, and acupuncture); (3) previous use of acupressure techniques; (4) allergy to tape; and (5) presence of acute back pain. 

### 2.2. Recruitment

Participants were recruited by flyers in primary care offices and clinics placed at the University of Pittsburgh Medical Center (UPMC) and the University of Pittsburgh campus. We received 31 self-referrals in response to the flyers. Participants who called the research office were screened for eligibility, and 24 participants who met the study criteria were scheduled for a research office visit. Three participants were not able to keep their weekly appointments. 

The study was conducted at the School of Nursing, University of Pittsburgh. During the participant's first clinic visit, informed consent and baseline assessments were obtained. Participants were randomly assigned with equal allocation to either the true or sham group using computer-generated simple randomization. After group assignment, participants received their first APA treatment. All of the participants received 4-weekly treatments. The first visit lasted from 1.5–2 hours, and the followup visits were approximately 30 minutes. All participants received free parking and a payment of $50 when the study was completed. The study participants were blinded regarding the group assignment (true and sham groups). The therapist (CHY) was not blinded. The University of Pittsburgh Institutional Review Board approved the study.

### 2.3. Acupoint Selection for True versus Sham APA

**Figure 1:**
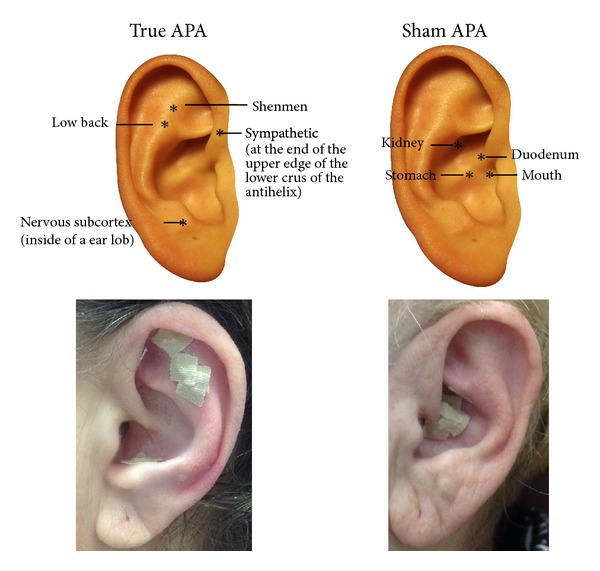
Acupoints for acupressure treatment.

The acupoints selected for true APA included three acupoints for alleviating stress and pain (i.e., shenmen, sympathetic, and nervous subcortex) and corresponding acupoints (low back). The sham acupoints selected were those which were located away from the areas of the ear where the participant was experiencing pain. The Chinese auricular map was used for acupoints selection [17]. Acupoints on participants in each group were identified using an electronic acupoint finder. The acupoint finder was connected to two probes: one was held by the participant; the other was used by the therapist to locate the acupoints. The acupoint was identified when the locator made a sound indicating the corresponding location on the body. Vaccaria seeds were carefully taped onto each selected auricular point. The therapist demonstrated the pressing technique to participants before asking them to do a reciprocal demonstration. Moderate stimulation was used for the therapy. Participants were told to press the seeds at least 3 times a day for 3 minutes at a time. They were also instructed to press the seeds for 3 minutes whenever they experienced pain. Participants were asked to remove the taped seeds by the end of the 5th day after seed placement so that the ear had no tape for two days of each week to let the ear points recover and restore sensitivity prior to the next treatment. Participants were told to contact the research office for seed replacement if the taped seeds fell out during the 5-day treatment.  Figure 1 shows the acupoints used for both groups and actual seed placement.

### 2.4. Measures 

#### 2.4.1. Brief Pain Inventory Short Form (BPI) [31]

The BPI was used to assess pain intensity (severity) and the impact of pain on functioning (inferences) on a 0–10 point numeric rating scale in the past 24 hours. BPI has established reliability and validity [31]. A 30% improvement was considered the threshold for identifying clinically meaningful improvement in pain intensity [32]. In this study, single score of “worst,” “average,” and “overall pain intensity” which was a composite of the four pain items (a mean severity score) was used in the final data analysis.

#### 2.4.2. Roland-Morris Disability Questionnaire (RMDQ) [33, 34]

The RMDQ, 24-item measure, was used to assess the impact of back pain on their daily functioning. The score ranged from 0 (no disability) to 24 (maximum disability). RMDQ is a reliable, valid, and sensitive measure [33, 34]. A clinically meaningful change on the RMDQ is 30% from baseline to the completed treatment [35].

#### 2.4.3. The Modified Oswestry Low Back Pain Disability Index (ODI) [36]

The ODI was used to measure a patient's impairment and quality of life on 10 items with 0–5 point scales [37]. The score ranges from 0–100%; a lower score indicates less disability. It has been used to establish disability level and stage of a patient's acuity status [36]. The minimal clinically important difference for the Oswestry is 6 points (8%) [38].

#### 2.4.4. Fear-Avoidance Beliefs [39]

 This was measured by a modified form of the Fear-avoidance beliefs questionnaire (FABQ) [39] that focuses on patient's beliefs about how physical activity (4 items) and work affects their pain (7 items) [39].

#### 2.4.5. The Pain and Catastrophizing Scale (PCS) [40]

The PCS was included to detect exaggerated and negative interpretations of pain. It is a self-report scale that consists of 13 items. Participants were asked to reflect on past painful experiences and to indicate to which degree he/she experienced symptoms such as helplessness or rumination when feeling pain. This is a 0–4 Likert scale (score sum 0–52) with responses ranging from “not at all” to “all the time,” and high scores indicate stronger catastrophizing.

#### 2.4.6. WHO Quality of Life-BREF (WHOQOL-BREF) [41]

The WHOQOL-BREF includes 26 items, self-administered, which measure the following broad domains: physical health, psychological health, social relationships, and environment. It was derived from WHOQOL-100, and the 26-items had established good reliability and validity [42].

#### 2.4.7. Treatment Satisfaction [20]

This is a 12-item scale and was used to assess the participants' satisfaction with the treatment and the extent to which they perceived the treatment to be a burden. The satisfaction form is a modified version of the satisfaction questionnaire used by our previous study of low back pain [20]. In our previous study, treatment satisfaction was sensitive to changes in pain intensity [20].

#### 2.4.8. Daily Diary

The daily diary included pain assessment (four items of pain intensity from the BPI, worst, average, right now, and least), intervention monitoring regarding APA practice (i.e., side effects, frequency, and duration of pressing on seeds), and medication use (including supplements). To monitor the safety of APA, participants were also asked about adverse events at each visit.

### 2.5. Data analysis

Descriptive statistics were used to present demographic characteristics and outcome measures. The equality of the mean change score from the baseline to the completion of the 4-week true or sham acupressure treatment was tested with the Wilcoxon-Mann-Whitney test [43]. The comparison of the proportion of clinically important difference (improved percentage >30% for pain intensity and RMDQ) between true and sham acupressure treatments was tested by Fisher's exact test [44]. Cohen's was used to calculate effects sizes [45]. The adherence rate of APA was used to determine the feasibility of participants practicing APA at home. The adherence rate was defined as participants who were able to follow at least two-thirds of the suggested pressing time (at least 2 times/day, 2 minutes/time). In order to analyze the patients' experiences of APA, content analysis was used to analyze the data collected from open-ended questions in the daily diary. All of the data analyses were performed using SAS software, version 9.2 [46].

## 3. Results

### 3.1. Demographic Characteristics

**Figure 2:**
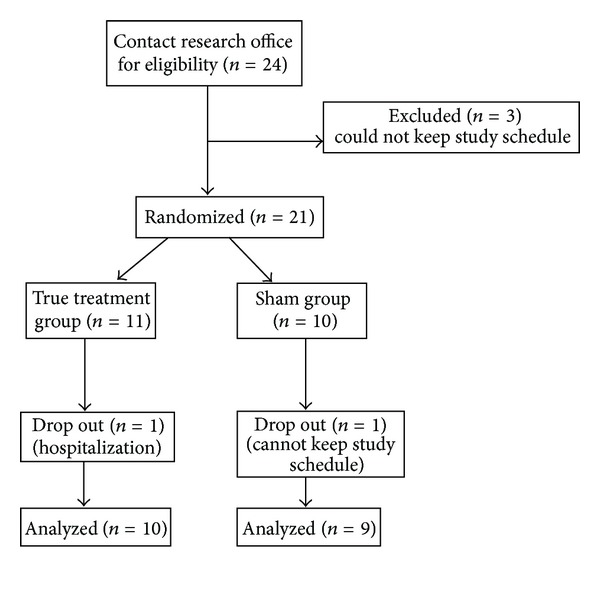
Flow chart of patient recruitment.

In total, 21 participants were randomized into the study (11 in true group and 10 in sham group), see Figure 2. Two subjects dropped out: one was due to hospitalization for low back; and the other was unable to attend appointments. The retention rate for the study was 90%.  Table 1 presents the demographic characteristics of the 19 participants who completed the study (10 in the true APA group and 9 in the sham APA group). The age of participants ranged from 20 to 70 years old. The majority of the participants were white (89%). 

### 3.2. Pain Intensity Change Pattern

**Figure 3:**
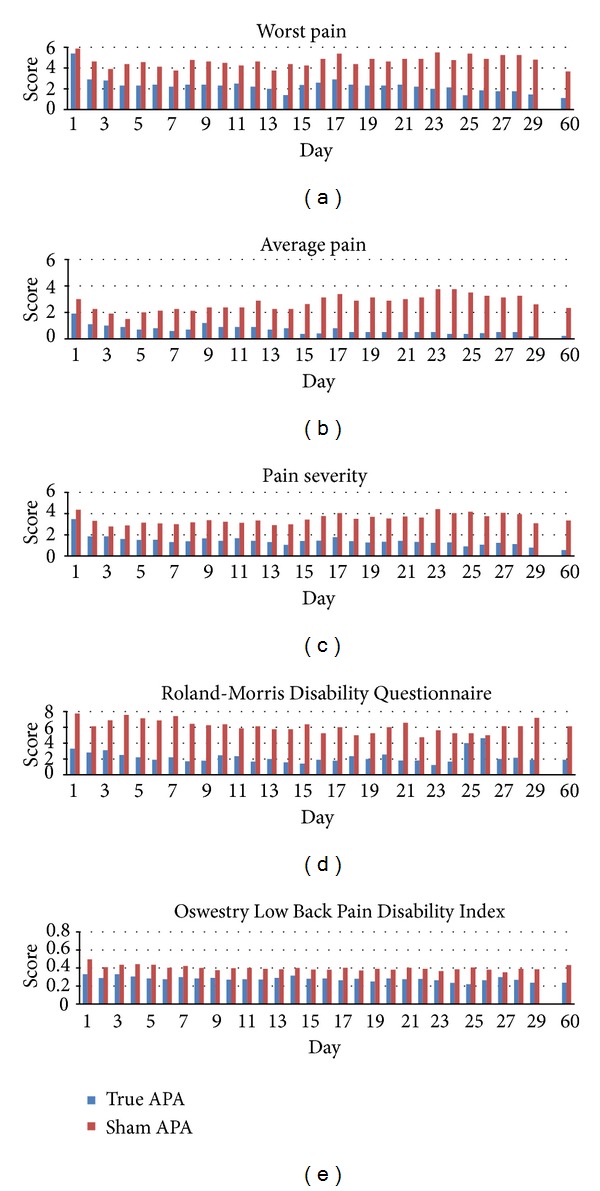
Pain intensity and back-specific disability change patterns for both groups.

Figure 3 illustrates the change in reported pain intensity (worst, average, and overall pain severity) reported in the daily diary from baseline to day 29 (completion of the APA treatment) and at the 1-month followup (day 60). For subjects in the true APA group, the worst pain score decreased 46% (2.5 points) (data not shown but is available upon request) from baseline to one day after APA treatment. This decrease was maintained or gradually decreased over the course of APA treatment. The scores of “average pain” and “overall pain severity” had a similar change in pattern. Participants in the true APA group had over 70% improvement in “worst pain” and “overall pain severity” after 4 weeks of APA treatment, while participants in the sham group only had 18% improvement in “worst pain” and 29% in “overall pain severity.” Table 2 shows the results for the pain intensity change for both groups. Participants in the true APA significantly improved at 4 weeks in worst and overall pain intensity than those in the sham group (*P* = 0.00 and 0.02, resp.). While the average pain measure was not significant, they were in the expected direction. Corresponding effects sizes were 1.50 for worst pain and 1.58 for overall pain intensity when compared to the mean differences between the completion of 4-week APA and baseline assessment for the true and sham groups. Participants in the true group had statistically significant clinical improvement in worst pain (*P* = 0.02) and overall pain intensity (*P* = 0.03) after a 4-week APA and one-month followup (Table 3). 

### 3.3. Back-Specific Disability Change Pattern

Back-specific disability change patterns (i.e., RMDQ and ODI) from the diary entries are displayed in Figure 3 for both groups. For the RMDQ, participants in the intervention group experienced a reduction in symptoms of 42% from baseline after completing a 4-week APA treatment and remained at the same improved percentage at one-month followup, while participants in the sham group had reduced symptoms 7% from baseline after completing a 4-week APA treatment and a 21% reduction at 1-month followup. While these findings were not significant, they were in the expected direction. The effect size for the RMDQ was 0.25. There was no significant difference between groups in proportion of subjects experiencing a clinically meaningful improvement of the RMDQ (defined ≥30% improvement).

For the ODI, participants in the true APA group had reduced symptoms of 28% from baseline after completing the 4-week APA treatment and 28% at 1-month followup, while participants in the sham group experienced a reduction in symptoms of 23% from baseline after completing the 4-week APA treatment and a 14% reduction at the 1 month followup. While these findings were not statistically significant, they were in the expected direction. 

### 3.4. Psychological Factors and Health Related Quality of Life

Table 2 shows the results of PCS (pain and catastrophizing), fear and avoidance and the subscales of health-related quality of life assessment at baseline, after 4 weeks of APA treatment and at 1-month followup. Participants at the completion of treatment reported an 86% reduction in catastrophizing for the true APA group and a 64% reduction in the sham group from baseline. Participants in both groups had similar percentage reductions in fear and avoidance beliefs for work and physical activity. The assessment of health-related quality of life did not change (≤7%) at each of the time points for both groups. While these findings were not statistically significant, they were in the expected direction. Effect sizes were 0.02.

### 3.5. Adherence Rate and Safety of APA Treatment

Table 4 presents the adherence rate of APA practice at home. The adherence rate was 93% at week one and gradually decreased to 88% at week four for true APA. Participants in the sham group had higher adherence rate than participants in the true APA. Participants in both groups reported that their ear had more sensitive sensation (*n* = 3, 16%), soreness (*n* = 4, 21%), and discomfort (*n* = 4, 21%) after the seed placement. This discomfort usually appeared on day 1~2 and gradually disappeared. Participants also reported itching (*n* = 7, 37%) and sleep disturbance when sleeping on the APA side (*n* = 2, 11%). Participants reported that compared to their back pain, the ear discomfort was tolerable.

### 3.6. Treatment Satisfaction

95% of participants believed that they were enrolled in the true APA group. All of the participants in both groups were satisfied with their care. For participants who received true APA 90% reported feeling better or much better, and 100% were satisfied with their care (Table 5). All of the subjects thought it was not difficult to press the taped seeds for 3 times/day and 3 minutes/time (Table 6). 

## 4. Discussion

This pilot RCT aimed to assess the feasibility, safety, and initial treatment effects of a 4-week protocol of APA to manage CLBP. To date, this is the first study to use APA to reduce CLBP under controlled conditions (i.e., compared to a sham group). The reductions of worst pain and overall pain intensity in true APA group were statistically greater than participants in the sham group. The retention rate for this study was 90%. There were few adverse effects reported by the participants. All of the participants felt it was not difficult to do APA pratice at home. This evidence indicates that a 4-week APA is feasible and potentially safe for CLBP. Before interpretation of the current study findings, several study limitations must be acknowledged. First, as this study was performed with limited funds, there was no research associate available to administer assessment instruments; rather the PI (CHY) and treating clinician administered the self-rating questionnaires. As she was unblinded, this introduces potential bias, although all of the measures were subject to self-rating and none were clinician-rated. Second, the small sample size limits the generalization of the study findings. A future study needs to consider other disease-specific measures related to low back pain (i.e., RMDQ) as the inclusion criteria, since physical disability is also a major outcome variable for CLBP [47]. Third, there is no empirical evidence regarding the specificity and sensitivity of electronic acupoint finder we used in the current study. Therefore, other methods to identify acupoints are suggested, including the participant's subjective complaint of symptoms which are corresponding to auricular points [48] and/or ear skin surface changes. Fourth, we did not collect information on the causes of CLBP. Given the preliminary findings in pain reduction, improved physical function, high adherence, high retention rate, and overall safety, APA has promising potential as an adjunct therapy for CLBP. Further study is needed to replicate and expand the current study design to a large-scale randomized clinical trial to determine the efficacy of APA treatment for CLBP.

Sham point selection and forms of stimulation are the key factors to treatment outcomes [49]. To date, there are few published clinical research studies that have suggested optimal criteria to select sham acupoints for auricular therapy. The selection of sham acupoints has been debated in the acupuncture studies targeting both body and auricular sites [50, 51]. Findings from this study show that true APA had a superior improvement of pain intensity and back-specific disability relative to subjects in the sham group suggesting that we had a credible sham comparison. Another pilot study has shown that true APA can reduce chemotherapy-related nausea and vomiting as compared to a sham group [52]. These preliminary findings are consistent with the meta analysis conclusions [51] suggesting that the effects of auricular therapy may have acupoint specificity, that is, the specific acupoints on the ear correspond to specific areas of the body. 

The form of stimulation is another key factor for treatment outcome [49]. APA was shown to have a similar effect to that of acupuncture on autonomic functioning favoring parasympathetic over sympathetic activity, with reduction in heart rate and increase in heart rate variability in 14 healthy volunteers [53]. APA, without using needles, offers a less invasive alternative to acupuncture and can be self-administered. Studies are still needed to investigate the stimulation “intensity” parameter of APA for treatment effects. Thus, a sham group with seeds but no pressure is needed to address this concern. 

The underlying mechanism of auricular therapy in treating disease/symptoms is still unclear. TCM has claimed auricular therapy as part of acupuncture for 2000 years. It states that the ear is related to all parts of the human body including each of the internal organs, and that all meridians have reference points on the ear. In TCM, a disease is considered to be caused by the imbalance of a person's energy, Qi [17]. The stimulation of auricular acupoints regulates Qi and activates the meridians and collateral systems [17]. If the mechanism of auricular therapy follows the meridians rationale of acupuncture, the studies of auricular therapy should bear similar placebo effects as those found in acupuncture [49, 54]. Our pilot findings indicate that true APA works better than the sham APA and suggest that the underlying mechanism of auricular therapy to treat disease/symptoms may be different from acupuncture theory. In 1950, the French neurosurgeon Paul Nogier theorized that the ear represents the inverted fetus within the womb [17, 18]. The whole anatomical body relation to auricular points in Chinese ear medicine has been modified according to Nogier's theory [17, 18]. However, it is clear that a better understanding of how APA reduces CLBP (i.e., biological mechanism) is required for the acceptance of APA in clinical practice. 

## 5. Conclusion

We found a 70% improvement in pain intensity from baseline (3.95 points for worst pain) after a 4-week APA treatment with maintenance of improvement at 1-month followup. This is greater than most studies in the acupuncture literature [50], which usually show 30% improvement [32]. Our current study duplicated our previous open trial findings [20], in which participants experienced an overall 45% reduction in pain intensity on day one after initiating APA treatment and 56% reduction in pain intensity at the end of the first week of treatment and reported even more improvement (73%) by the end of the 4-week treatment. In addition, these study findings also indicated a 42% reduction of RMDQ from baseline in the true APA group, which is better than the literature-suggested 30%, defined as “clinical improvement” [35]. More importantly, participants in this study received only four treatment sessions, while most acupuncture treatments need 6–12 sessions to achieve the greatest benefits. In order to address the stimulation “intensity” parameter of APA for treatment effects, a sham group with seeds but no pressure is needed.

## Figures and Tables

**Table 1:** Demographic characteristics of the participants.

	Mean (SD) or *n* (%)	
	True	Sham
Age	45.4 (21.8)	49.8 (14.4)
Gender		
Male (*n*)	2 (2%)	2 (22%)
Female (*n*)	8 (8%)	7 (78%)
Marital status		
Married/partnered (*n*)	5 (50%)	5 (56%)
Divorced/separated/other (*n*)	5 (50%)	4 (44%)
Education		
Primary (*n*)	1 (10%)	0 (0%)
Secondary (*n*)	2 (20%)	1 (11%)
College and above (*n*)	7 (50%)	8 (89%)
Ethnicity		
White (*n*)	9 (90%)	8 (89%)
Black (*n*)	1 (10%)	1 (11%)
Pain medication use at baseline		
Yes	2 (20%)	4 (44%)
No	8 (80%)	5 (56%)

**Table 2:** Effects of acupressure on pain, disability, psychological function, and quality of life.

Outcome measure (possible range)	Group	Baseline Mean ± SD	4 weeks Mean ± SD	4 weeks % change	*P* value^‡^	1-month followup Mean ± SD	1 month % change
Pain intensity							
Worst pain	True	5.40 ± 0.97	1.60 ± 1.71	−70	0.00	1.40 ± 1.43	−74
(0–10)	Sham	5.88 ± 1.89	4.38 ± 1.06	−26		4.14 ± 1.86	−29
Average pain	True	3.40 ± 0.70	1.15 ± 1.56	−66	0.39	0.80 ± 0.92	−76
(0–10)	Sham	5.13 ± 1.36	2.88 ± 1.13	−44		4.00 ± 1.29	−22
Overall pain intensity	True	3.48 ± 0.74	0.86 ± 0.79	−75	0.02	0.68 ± 0.79	−81
(0–10)	Sham	4.36 ± 1.28	3.09 ± 1.18	−29		3.61 ± 1.15	−17
Back-specific disability							
RMDQ	True	3.30 ± 2.54	1.67 ± 1.32	−49	0.82	1.90 ± 1.66	−42
(0–24)	Sham	7.75 ± 6.23	7.00 ± 6.74	−10		6.13 ± 5.28	−21
ODI	True	0.33 ± 0.04	0.23 ± 0.07	−31	1.00	0.24 ± 0.05	−28
(0-1)	Sham	0.50 ± 0.19	0.40 ± 0.14	−20		0.43 ± 0.16	−13
Psychological factors							
PCS	True	9.50 ± 5.72	1.33 ± 2.06	−86	0.25	2.90 ± 4.79	−69
(0–52)	Sham	17.63 ± 9.50	6.38 ± 6.84	−64		5.86 ± 7.03	−67
Fear avoidance beliefs							
Physical activity	True	11.25 ± 4.92	6.56 ± 7.38	−24	0.37	7.90 ± 6.38	−30
(0–24)	Sham	13.00 ± 7.16	10.14 ± 7.69	−22		8.43 ± 7.23	−35
Work	True	11.75 ± 9.35	5.44 ± 7.26	−54	0.85	6.60 ± 9.06	−44
(0–42)	Sham	18.86 ± 14.28	9.00 ± 8.15	−52		12.86 ± 13.06	−32
Health related quality of life							
Physical	True	12.63 ± 1.06	12.74 ± 1.75	1	0.24	12.74 ± 1.32	1
(0–100)	Sham	12.64 ± 1.77	13.43 ± 1.43	6		13.57 ± 1.49	7
Psychological	True	14.67 ± 1.78	14.13 ± 1.85	−4	0.74	14.40 ± 1.92	−2
(0–100)	Sham	14.58 ± 1.80	14.50 ± 1.77	−1		14.75 ± 2.32	1
Social	True	15.87 ± 2.91	15.07 ± 3.61	−5	0.59	15.80 ± 2.95	0
(0–100)	Sham	16.00 ± 2.76	16.50 ± 3.56	3		16.83 ± 3.70	5
Environment	True	16.30 ± 2.84	15.60 ± 3.01	−4	0.33	15.75 ± 3.01	−3
(0–100)	Sham	16.25 ± 2.48	16.06 ± 1.52	−1		16.69 ± 2.49	3

% change = (mean at 4 weeks − mean at baseline)/mean at baseline.

RMDQ: Roland-Morris Disability Questionnaire.

ODI: Modified Oswestry Low Back Pain Disability Index.

PCS: The pain and catastrophizing scale.

^‡^ *P* value is calculated by the Wilcoxon-Mann-Whitney test for the null hypothesis: *M*_*t*_ = *M*_*s*_.

**Table 3:** Summary statistics for clinically improvement difference in pain intensity and back-specific disability by treatment groups.

Outcome measure	Group	Change at completion of 4-week APA		P*	Change of 1-month followup		*P*
		≥30% (*n*)	<30% (*n*)			≥30% (*n*)	
Pain intensity							
Worst pain	True	9	1	0.0198	10	0	0.0108
	Sham	3	6		4	5	
Average pain	True	8	2	0.6285	10	0	0.0108
	Sham	6	3		4	5	
Overall pain intensity	True	10	0	0.0325	10	0	0.0108
	Sham	5	4		4	5	
Back-specific disability							
RMDQ	True	7	3	0.3698	6	4	0.6563
	Sham	4	5		4	5	

*P: *P* value obtained by Fisher's exact test.

**Table 4:** Adherence behaviors by week.

	Week 1	Week 2	Week 3	Week 4
Adherence				
True	93%	88%	88%	88%
Sham	98%	98%	96%	96%

**Table 5:** Satisfaction of auricular point acupressure treatment for pain.

	True	Shame
	*N* (%)	*N* (%)
Fewer episodes pain		
Yes	9 (90%)	6 (67%)
No	1 (10%)	3 (33%)
Pain improved		
Yes	10 (100%)	8 (89%)
No	0 (0%)	1 (11%)
Take less medication than before treatment		
Yes	6 (60%)	7 (78%)
No	1 (10%)	2 (22%)
Did not respond	3 (30%)	0 (0%)
Overall feeling		
Much better	5 (50%)	1 (11%)
Better	4 (40%)	6 (67%)
About the same	1 (10%)	2 (23%)
Worse	0 (0%)	0 (0%)
How much better mean (%) (SD)	54 (39.72)	45.83 (35.84)
Satisfaction about the progress		
Completely	7 (70%)	2 (22%)
Somewhat	3 (30%)	7 (78%)
Not satisfied	0 (0%)	0 (0%)

**Table 6:** Perceived burden for auricular point acupressure practice.

	True	Shame
	*N* (%)	*N* (%)
Frequency to pressure the taped seeds		
Not difficult at all	6 (60%)	3 (33%)
A little difficult	1 (10%)	4 (45%)
Do not think it is difficult	3 (30%)	2 (22%)
Duration to press the taped seeds		
Not difficult at all	7 (70%)	5 (56%)
A little difficult	2 (20%)	2 (22%)
Do not think it is difficult	1 (10%)	2 (22%)
